# Occupational and Lifestyle Factors of Male and Female Infertility Patients: Do They Impact ART Success?

**DOI:** 10.3390/medicina62061132

**Published:** 2026-06-10

**Authors:** Jelena Micić, Mladen Andjić, Jelena Dotlić, Katarina Ivanović, Aleksandar Trklja, Jovana Plešinac, Maja Maslovarić, Bojana Mihajlović, Lela Šurlan, Isidora Protić, Lidija Tulić, Jovan Bila, Jelena Stojnić

**Affiliations:** 1 Clinic for Gynecology and Obstetrics, University Clinical Center of Serbia, Dr Koste Todorovica 26, 11000 Belgrade, Serbiamaja.maslovaric@gmail.com (M.M.); lela.surlan@gmail.com (L.Š.); ivfjelena@gmail.com (J.S.); 2 Faculty of Medicine, University of Belgrade, Dr Subotića 8, 11000 Belgrade, Serbia

**Keywords:** lifestyle, diet, tobacco and alcohol, infertility, assisted reproduction success

## Abstract

*Background and Objectives*: Numerous risk factors for both female and male fertility have been established including age, ovarian reserve, infertility cause, occupational and lifestyle factors. The objective of our study was to determine the influence of occupational and lifestyle factors on assisted reproduction (ART) outcomes at a Serbian referral tertiary center. *Materials and Methods*: The study included all consecutive infertile couples undergoing ART at the Clinic for Ob/Gyn University Clinical Center Belgrade, from January 2019 to January 2022. Inclusion criteria comprised primary and unexplained infertility, age ≤ 45 years, body mass index ≤ 30 kg/m^2^ and undergoing fresh autologous ART cycles. All patients filled in the socio-epidemiological questionnaire that analyzed their lifestyle and habits. Medical history data and data regarding the current ART cycle were taken from patient records. The primary outcome was clinical pregnancy. *Results*: Our study included 501 couples (women and men) with infertility undergoing ART. Clinical pregnancy was achieved in 22.2% of examined patients. Achieving clinical pregnancy in the ART cycle for women was associated with younger age and use of vitamins, minerals, and trace elements, whereas younger age and absence of chronic illnesses were the most important factors for male partners. When women and men were assessed together as couples, achieving clinical pregnancy correlated only with the use of vitamins, minerals and trace-elements by both partners. *Conclusions*: This study confirmed that some occupational and lifestyle factors were associated with clinical pregnancy after ART in patients with unexplained primary infertility and normal BMI.

## 1. Introduction

Infertility, defined as the inability to achieve pregnancy after 12 months of regular unprotected sexual intercourse, affects nearly 15% of couples worldwide [1]. It has been estimated that approximately 85% of infertile couples have identifiable causes of infertility, such as ovulatory dysfunction, tubal disease, or male-factor infertility, while about 15% have unexplained or idiopathic infertility despite the use of current diagnostic techniques [1,2]. To address various forms of infertility, different methods of assisted reproductive technology (ART) have been developed in recent decades. These procedures include intrauterine insemination (IUI), in vitro fertilization (IVF), intracytoplasmic sperm injection (ICSI), the use of donor gametes, embryo freezing, and surrogacy [1,2].

Numerous risk factors for both female and male fertility have been established, including female age, ovarian reserve, and the cause of infertility. Assisted reproduction success rates vary from 20% to 35% per cycle, with success heavily influenced by patient characteristics and overall health, stimulation and cycle characteristics, and embryo quality [1,2]. Increasing evidence has shed light on the role of modifiable occupational and lifestyle factors among infertile couples in ART outcomes [3,4,5]. Occupational risk factors, such as radiation, toxic chemicals, vibration, and extreme temperatures, as well as lifestyle factors and nutritional status (i.e., tobacco use, alcohol consumption, and fruit and vegetable intake), are known to be critical determinants of normal reproductive function [3,4,5]. However, the mechanisms through which different toxic agents affect infertility are still under investigation, and recent studies have reported conflicting findings regarding the influence of occupational and lifestyle factors on ART outcomes [6,7,8].

Given the lack of data on occupational and lifestyle factors and their relationship to ART outcomes in the Serbian population, the objective of our study was to determine the influence of occupational and lifestyle factors among infertile couples on ART outcomes at a Serbian referral tertiary center.

## 2. Materials and Methods

The present study included all consecutive infertile couples undergoing ART at the Clinic for Gynecology and Obstetrics, University Clinical Center of Serbia, Belgrade, from January 2019 to January 2022. The inclusion criteria were primary infertility, unexplained infertility, and planned treatment with fresh autologous IVF/ICSI cycles. Moreover, inclusion criteria for female partners were age up to 45 years and body mass index (BMI) up to 30 kg/m. The exclusion criteria were known causes of male and female infertility. In men, these included sperm abnormalities, such as oligoasthenospermia and teratospermia. In women, they included fallopian tube obstruction, premature ovarian failure, endometriosis, polycystic ovary syndrome, uterine anomalies, myomas distorting the endometrial cavity, endometrial polyps, previous ovarian or tubal surgeries, hypothalamic amenorrhea, galactorrhea, and systemic or acute infectious diseases.

All patients were thoroughly investigated with basic and advanced infertility tests prior to ART (women—hormone assessments, blood count, coagulation testing, biochemical analyses, ultrasound examination, ovulation tracking, histerosalpingograpy, diagnostic hysteroscopy and laparoscopy, and genetic screenings; men—semen analysis and genetic screening). Unexplained infertility was considered in patients in whom, after all performed tests, reproductive specialists still could not pinpoint a biological obstacle for pregnancy. Primary infertility was diagnosed for patients that have never achieved any pregnancy.

All patients, both women and men, completed a socio-epidemiological questionnaire that assessed their lifestyles and habits. The questionnaire included employment status (categorized as unemployed, working indoors, or working outdoors); occupational hazards (including radiation, toxic chemicals, vibration, and temperature); active tobacco smoking; alcohol consumption (completely no vs. yes that stands for at least one standard drink for both women and men) and frequency of drinking (categorized as occasional or regular/daily); consumption of carbonated and energy beverages; use of dietary supplements, i.e., multivitamin products containing vitamins (C, E, A, D, B1, B2, B3, B5, B6, B12 and folic acid), minerals, and trace elements (Ca, Fe, Zn, Cu, Mg, Mn, Mo, F, Cr, Se, and I) once per day, regularly every day at least for the previous three months; regular physical activity (defined according to World Health Organization Physical Activity Guidelines as moderate-intensity, i.e., mild aerobic exercise for at least 2 h per week); and duration of the current relationship, measured in months.

Medical history data were obtained from patient records, including age, BMI, family and personal history of chronic and severe illnesses, duration of cohabitation and infertility (months), gynecological history for women (cycle, parity, and previous ART), and data regarding the current ART cycle. ART cycle outcomes were considered as successful, defined as achievement of pregnancy, or unsuccessful if no pregnancy was achieved. The number of embryos and the outcomes of the achieved pregnancies, including ectopic pregnancy, miscarriage, and delivery, were also recorded.

The primary outcome was ART success presented as achieving clinical pregnancy (a gestational sac in the uterus with a viable embryo confirmed by ultrasound). In addition, as all achieved clinical pregnancies were regularly followed and controlled, we also presented their outcomes (miscarriage or childbirth; gestational week of delivery, and birth-weight of children).

Detailed descriptions of the stimulation protocol, patient monitoring, oocyte retrieval, and embryo transfer have been provided in previous studies [9,10]. The patients were informed about all therapeutic procedures during ART, the obstetrical management of achieved pregnancies, and the study itself, after which they all provided written informed consent. The Institutional Review Board of the University Clinical Center of Serbia approved the study (Decision No. 330/5; 26 March 2026).

Data were statistically analyzed by SPSS 20 for Windows software Version 20 and using standard methods of descriptive (frequency, percent, mean, and standard deviation) and analytical statistics. To compare patient characteristics ANOVA and Kruskal–Wallis χ^2^ tests were applied. Spearman’s correlation was performed to investigate the associations of lifestyle and occupational factors of women and men undergoing ART. Finally, binary logistic regression was applied to construct models of associations of investigated occupational and lifestyle factors (independent variables) with achievement of clinical pregnancy (dependent variable assessed in binary mode as yes vs. no). The analysis was performed for women and men first separately and then together (whole sample and as the behavior of couples). Therefore, three databases (for women, men, and couples) were formed. In the couples database, lifestyle parameters were categorized as (1) if neither of the partners had the characteristic, (2) if one of the partners had the characteristic and (3) if both partners had the characteristic. Moreover, as observations for women and men are correlated if they have the same pregnancy outcome as a couple, to avoid violation of independence of observations, we also performed mixed-model analysis.

## 3. Results

This study included 501 infertile heterosexual couples undergoing ART. The rate of achieving clinical pregnancy after ART according to the current literature and the patients’ age range (18–40) should be 37.5%. Consequently, with this sample size and the alpha error 0.05, the study power was >98%.

Table 1, Table 2, Table 3, Table 4 and Table 5 present the socio-epidemiological and medical parameters, as well as the lifestyle characteristics, of the infertile couples. The women were 23–45 years old, and the men were 27–59 years old. Male patients were significantly older than female patients (*p* = 0.001). In our sample, 18.8% of the women and 74.7% of the men were overweight. Couples reported having experienced infertility problems for 1 to almost 20 years. Among the investigated couples, 65.8% had already undergone ART for infertility treatment. Our patients were generally healthy, although women reported chronic illnesses significantly more often than men (*p* = 0.001).

Among the investigated patients, 82.3% of the women and 88.0% of the men were employed. Only 5.8% of the couples had both partners unemployed. Most women and men worked indoors in office settings and had no occupational hazards. Only three couples reported that both partners had occupational risk factors for fertility. Nevertheless, significantly more men than women worked outdoors.

Although the majority of the patients in our sample did not drink alcohol, around 20% of the couples were occasional alcohol consumers. Significantly more men than women drank alcohol (*p* = 0.001) and consumed carbonated drinks (*p* = 0.047). Less than 1% of the patients consumed energy drinks. However, men consumed energy drinks almost twice as often as women did (*p* = 0.001). Most investigated women and men reported using vitamins, minerals, and trace elements (66.7% of couples), although significantly fewer men than women used them (*p* = 0.001).

In our sample, the majority of both women and men were nonsmokers. However, in 10.6% of couples, both partners were smokers. Few examined patients engaged in regular physical activity. Only 3.4% of couples reported regular physical activity for both partners. Nevertheless, no significant differences were observed between women and men.

In our study, clinical pregnancy after ART was achieved in 111 patients (clinical pregnancy rate 22.2%). However, 25 pregnancies ended in miscarriage, generally in the second month of pregnancy, and one pregnancy was ectopic. Overall, 17% of the patients delivered a healthy child, mostly by cesarean section (72.9%).

Pregnancy was achieved significantly more often among patients (both women and men) who were younger, when both or at least one partner used vitamins, minerals, and trace elements, and when neither partner drank alcohol. However, unhealthy habits, defined as smoking and drinking, had no significant impact on pregnancy achievement among the investigated women and men (*p* = 0.071). Moreover, having an unhealthy lifestyle had no significant impact on pregnancy achievement (*p* = 0.122).

When alcohol consumption and cigarette smoking were assessed together for each patient, 50.5% of the investigated women and men had unhealthy habits. Men smoked cigarettes and drank alcohol significantly more often than women (*p* = 0.001). Finally, when alcohol consumption, cigarette smoking, carbonated and energy drink consumption, and lack of regular recreational activity were assessed together, 68.3% of the investigated women and men had unhealthy lifestyles. Men had an unhealthy lifestyle significantly more often than women (*p* = 0.001).

Correlations between lifestyle and occupational factors in the overall sample of women and men undergoing ART are presented in Table 6. The BMI was higher in patients who were unemployed, had environmental risk factors, had chronic illnesses, drank alcohol and carbonated drinks, and did not use vitamins. Patients who smoked also drank carbonated drinks and generally did not engage in regular physical activity. Alcohol consumption was associated with energy drink consumption and the absence of chronic illnesses. Patients who used vitamins generally also engaged in regular physical activity. In addition, having chronic illnesses was correlated with vitamin use, male sex (*p* = 0.001), and an unhealthy lifestyle, defined as alcohol consumption, cigarette smoking, carbonated and energy drink consumption, and a lack of regular recreational activity (*p* = 0.024). It can be seen that some behaviors correlated and were registered together in patients. Correlations were generally logical and in accordance with the lifestyle (healthy or not healthy). Still, no investigated behavior was associated with all other behaviors.

For regression models the standard rule of thumb is to have at least 10 events per variable. In our models, 11 variables, i.e., occupational and lifestyle parameters of investigated patients, were tested (11 × 10 = 110). As the clinical pregnancy rate was 22.2%, the sample size for reliable regression analysis should be 110/0.222 = 495.5 patients. After confirmation of sample size adequacy, we performed the regression analysis.

Regression analysis showed that the predictors of achieving pregnancy in the ART cycle for women were younger age and use of vitamins, minerals, and trace elements, whereas younger age and absence of chronic illnesses were the most important factors for male partners (Table 7). Still, the significance of male health was borderline. Moreover, because the lower limit of confidence interval is close to the zero, with a very wide range of confidence interval, we have to acknowledge that a high degree of uncertainty remains regarding the exact effect size of the impact on achieving clinical pregnancy after ART by the male health status in our population. One potential reason for such unusual results may come from the fact that men had significantly fewer chronic illnesses than women, which is generally unexpected. However, it can be a particularity of the study sample or one of the unexplained but underlying causes of infertility among the investigated women. These findings should therefore be confirmed in future studies on different samples.

When women and men were assessed together (whole sample) and as couples, achieving clinical pregnancy was associated only with younger age and use of vitamins, minerals, and trace elements by both partners for at least three months before ART (Table 8). In addition, according to the performed mixed-model analysis with restricted maximum likelihood estimator, fixed effects were evaluated using Type 3 tests. The obtained Akaike’s Information Criterion was 1089,726 and the Bayesian Criterion was 1,109,365. Out of all investigated lifestyle characteristics (fixed effects), only the use of vitamins, minerals, and trace elements was proven to impact the achievement of clinical pregnancy after ART (*p* = 0.011).

## 4. Discussion

Maternal age has been established as a major predictive factor of ART outcomes. The literature shows that women aged ≤31 years have significantly higher pregnancy and live birth rates, whereas after the age of 40 years, the success rate declines drastically [9]. In addition, it was recently shown that male fertility potential declines significantly with advancing age. Consequently, paternal age can also significantly influence ART outcomes [10,11]. Our study results confirmed that younger age in both partners was associated with a significantly higher chance of achieving pregnancy through ART, regardless of lifestyle or other socio-epidemiological characteristics.

It is well known that different chronic conditions in women can negatively impact achieving clinical pregnancy after ART by influencing implantation and overall fertility. Several studies have documented that women with type 2 diabetes have a lower chance of achieving clinical pregnancy after ART, while the risk of miscarriage is 3.3 times higher compared with healthy patients [12]. Other endocrinological conditions, such as thyroid disorders and polycystic ovary syndrome, can cause hormonal imbalances that interfere with ovulation, oocyte quality, and implantation [12]. Autoimmune disorders can cause the immune system to attack the embryo, leading to implantation failure or miscarriage [13]. The presence of comorbidities in male partners, and their impact on ART outcomes, has also been demonstrated in the available literature. Men with hypertension were found to have significantly worse semen parameters, including lower ejaculate volume, lower total sperm concentration, reduced numbers of progressively motile spermatozoa, and a lower total number of motile spermatozoa [14,15,16]. In our study, interestingly, achieving clinical pregnancy after ART was associated only with the absence of comorbidities in male partners. However, this finding requires further analysis in larger populations, as comorbidities were more frequent in men than in women in our sample.

Numerous studies in the literature have shown that women with a BMI ≥ 35 kg/m^2^, and especially those with a BMI ≥ 40 kg/m^2^, had a significantly lower number of normally fertilized oocytes, while the odds of achieving clinical pregnancy were approximately 50% lower compared with women of normal weight [12]. In addition, previous investigations demonstrated that in ART cycles involving obese male partners, significantly lower clinical pregnancy rates were obtained compared with procedures involving men of normal weight, although no significant difference in embryo quality was observed with regard to male partner BMI [17]. Nevertheless, our investigation did not prove that a higher BMI in the female partner negatively affected achieving clinical pregnancy after ART. The reason for such findings could be the fact that all investigated women had adequate BMI as an inclusion criterion for both the ART procedure and the study.

Based on previously published data, it could be expected that the dietary habits of the female partner, and to some extent those of the male partner, could affect ART outcomes. Data from the literature indicate that following a Mediterranean diet while planning pregnancy improves live birth and pregnancy rates after ART, while various healthy diets improve both ART outcomes and natural conception rates [18]. One potential reason could be that the Mediterranean diet and vegetable consumption are associated with higher levels of AMH, while a high intake of red meat and carbonated beverages can decrease AMH levels [19]. However, significant variability in the components of healthy diets has been observed across studies. In addition, investigations have shown that patients who regularly had breakfast during the last week before the ART procedure more often achieved pregnancy after ART and had higher live birth rates and lower miscarriage rates [20]. Nevertheless, in some previous studies, women’s intake of sugar and artificially sweetened beverages, as well as greater intake of vegetables, fruits, whole grains, legumes, and fish, was not correlated with success in infertility treatment with ART. These findings correspond with the results of our study, as we did not establish any association between specific dietary habits and ART outcomes [21,22].

One of our main findings is that the use of dietary supplements (vitamins, minerals, and trace elements) in both women and men was associated with improved ART outcomes. These findings are consistent with the literature, which confirms that supplementation can enhance gamete quality, reduce oxidative stress, and increase the chances of clinical pregnancy and live birth [23]. Some randomized controlled trials have reported that antioxidant supplementation in men with infertility issues may increase the live birth rate and clinical pregnancy rate [23]. Data on the importance of supplementation with the active form of folic acid showed that an intake of 800 µg of folic acid or more per day, compared with 400 µg or less, significantly increased the chance of live birth and fertilization rates while reducing the risk of cycle failure before embryo transfer [24]. A systematic review highlighted the use of coenzyme Q10 and L-carnitine supplementation and their positive effects on sperm concentration and motility, although there is currently no clear evidence that these effects consistently translate into higher pregnancy and live birth rates [25]. Likewise, studies have shown that folic acid, B-complex vitamins, and healthier dietary patterns are associated with better outcomes in ART procedures [26].

Previous research highlighting the impact of lifestyle on infertility has reported that chronic oxidative stress resulting from unhealthy behaviors, such as smoking and alcohol consumption, negatively affects IVF outcomes. When behaviors of couples with infertility (women and men together) were assessed, out of all investigated lifestyle characteristics, alcohol consumption (with both partners being occasional drinkers) was the only one that correlated with ART outcomes.

Alcohol is notable for its toxicity and harmful effects on both overall and reproductive health. During alcohol metabolism, high levels of reactive oxygen species, including free radicals, are formed; these can react with numerous other molecules, causing mutations and even cell death [27,28]. Published data show that weekly consumption of >84 g of alcohol reduced the chances of achieving pregnancy after ART by 7% if women were alcohol consumers, while live birth rates after ART decreased by 9% if male partners drank alcohol during ART cycles [29]. Finally, if both partners consumed alcohol, the probability of live birth after ART was significantly lower compared with couples in which the partners did not drink alcohol [29]. These findings correspond with the results of the correlation analysis performed in our study and indicate that the detrimental effect of alcohol on reproduction and achieving clinical pregnancy is more pronounced if both partners drink alcohol. However, the relationship between alcohol intake and ART outcomes remains uncertain, as different studies have reported conflicting results. Some investigations in the general population could not confirm the adverse effects of moderate drinking on fertility [30]. In our study, the level of significance of the effect that alcohol can have on achieving pregnancy after ART was also marginal, and consequently this finding was not confirmed by the regression analysis. Therefore, further studies on this matter are still needed.

Cigarettes contain a large number of toxins and carcinogens, such as tar, carbon monoxide, nicotine, and heavy metals, all of which can have detrimental effects on general health and cause the deregulation of the reproductive and hormonal systems, lowering the chance of a successful and healthy pregnancy [31]. In women, nicotine has an antiestrogenic effect, leading to an increased ratio of androgens to estrogens. Tobacco ingredients can interfere with the secretion of male reproductive hormones, compromise spermatogenesis, cause structural abnormalities in sperm, and worsen overall sperm quality [32]. However, published data regarding the effects of cigarette smoking on reproduction in women and men are conflicting. In some studies, after adjusting for relevant confounders, smoking status was not significantly associated with achieving clinical pregnancy or with live birth rates in patients undergoing ART [33]. Moreover, smoking intensity (mild/moderate/heavy; occasionally/daily) was also not correlated with ART outcomes [34]. These findings correspond with the results of our study.

A review of the available literature revealed that only a limited number of studies have investigated the impact of the workplace environment, such as open versus closed spaces, on achieving clinical pregnancy after ART, which adds further value to our findings. Existing studies mainly address the negative impact of exposure to pesticides and aromatic solvents on male fertility, including reduced sperm concentration, quality, and motility [35].

Chronic inflammatory conditions such as endometriosis and some autoimmune diseases are well known causes of infertility as the inflammatory process affects follicular dynamics and ovulation. Increased plasma concentrations of inflammatory markers have also been shown in polycystic ovary syndrome. The immunoproteasome is formed after proteasome activation by proinflammatory cytokines, and their role is to remove proteins that are malformed or damaged by stress conditions or that need to be degraded by standard turnover. Further studies could investigate the potential role of inflammatory factors as well as proteasome and immunoproteasome determinations in infertility patients, specifically those exposed to occupational and environmental risk factors or those who have an unhealthy lifestyle. Both tobacco smoking and chronic alcohol consumption promote systemic inflammation. The high levels of reactive oxygen species and free radicals, originating from cigarette smoke or generated during the metabolic breakdown of alcohol, cause direct cellular damage, prompting a constant inflammatory repair cycle [36,37,38]. The current study was observational in design, but future research could investigate more thoroughly the molecular basis of unexplained infertility in regards to patients’ lifestyle.

Finally, it should be noted that this study has some limitations. First, we enrolled a highly selected population of couples with either primary infertility without identifiable cause or truly unexplained infertility. Such strict sampling methodology prevents generalizability to patients with other infertility causes. Further studies should investigate the impact on achieving clinical pregnancy after ART according to different known infertility causes. Moreover, the observational design of the study, self-reported questionnaire data and no randomization, prevented the establishment of causation. Residual confounding is highly likely. Another potential limitation coming from the sampling methodology is the healthy-user bias. Infertility patients are prone to engaging in different health-conscious behaviors in order to maximize their chances for achieving clinical pregnancy after ART. However, although the majority of investigated patients did not smoke or drink alcohol, few of them took food supplements and had regular physical activity. Therefore, we considered that healthy-user effects on our findings were not significant.

Another limitation lies in the applied questionnaire and the construct of its items. Specifically, the socio-epidemiologic questionnaire, though in everyday use, was not validated. Moreover, the lack of quantifiable data should be pointed out. The questionnaire investigated the use and the frequency of tobacco, alcohol, dietary supplements, and carbonated and energy beverage consumption, while their amount was not in focus. This aspect is planned to be assessed in further studies. Moreover, the data such as adherence to dietary supplements was self-reported. Still, we believe that all of our patients reported data truthfully.

Furthermore, our regression analysis took into consideration only lifestyle factors while very important factors for achieving clinical pregnancy after ART like the stimulation protocol, gonadotropin dose, number of retrieved and mature oocytes, use of IVF vs. ICSI, embryo quality, day of transfer, number of transferred embryos, and embryo stage were not included. We opted for such models as all ART procedures are preformed according to current protocols, and medical parameters that can impact the ART outcome are well established. Consequently, this study and the regression analysis only investigated the impact of occupational and lifestyle factors.

Finally, the data presented in Table 2—age, BMI and number of IUI, IVF and miscarriages—were normally distributed while the duration of relationships and infertility were not. However, for clarity and simplicity of data presentation, we opted to present all of these parametric data with mean +/− SD while their differences were assessed by ANOVA. In this study, no adjustments for multiple comparisons across the separate regression models were made as this is planned to be performed in future research. Moreover, in most countries, healthcare systems support ART for women up to 40 years while our country allows ART up to the age of 45 at the expense of the state. Therefore, in worldwide studies, the age range is generally 18–40 and that is the age group with which we compare our findings in all investigations as well as the presented one.

## 5. Conclusions

The results of the present study confirmed that some occupational and lifestyle factors were associated with clinical pregnancy after ART in patients with unexplained primary infertility and normal BMI. Achieving clinical pregnancy in such patients correlated with younger age of patients and the use of vitamins, minerals, and trace elements. In addition, the absence of alcohol consumption by both partners for at least three months prior to ART might have a positive effect on ART outcomes in some cases. Therefore, couples undergoing ART due to infertility should be counseled about the impact of modifiable factors, such as lifestyle, on treatment outcomes. Until otherwise confirmed, patients with unexplained primary infertility and normal BMI should be encouraged to comply as much as possible with a healthy diet, food supplementation, and alcohol abstinence, at least during the ART cycle, as these behaviors are not only good for general health but might be related to increased chances of successful conception after fertility treatment.

## Figures and Tables

**Table 1:** Descriptive data of investigated couples with infertility and ART outcomes.

Parameters	Minimum	Maximum	Mean	Standard Deviation
Age of females	23.00	45.00	37.27	4.42
Age of males	27.00	59.00	39.57	5.57
BMI of females	16.49	30.00	23.05	3.05
BMI of males	19.00	43.29	27.49	3.74
Cohabitation in months	1.00	270.00	82.19	46.41
Infertility in months	12.00	236.00	62.61	42.72
Number of IUI before	0.00	8.00	1.04	1.31
Number of IVF before	0.00	6.00	1.33	1.23
Number of miscarriages	0.00	2.00	0.26	0.66
Miscarriage month	1.00	4.00	1.61	0.81
GW of delivery	23.00	40.00	37.46	2.67
Weight of child 1	900.00	4450.00	2994.44	634.41
Weight of child 2	1730.00	2910.00	2325.41	359.48
Parameters			Frequency	Percent
ART outcome	no		390	77.8
	ectopic		1	0.2
	miscarriage		25	5.0
	delivery		85	17.0
Clinical pregnancy	one child		86	77.5
	twins		25	22.5
Delivery type	vaginal		23	27.1
	CS		62	72.9

**Table 2:** Descriptive data of examined couples with infertility according to ART outcome.

Parameters	No Pregnancy		Clinical Pregnancy		Between-Group *p*
	Mean	Standard Deviation	Mean	Standard Deviation	
Age	38.72	5.22	37.35	4.78	0.001
BMI	25.29	4.12	25.18	3.89	0.704
Relationship in months	83.31	48.62	78.34	37.09	0.158
Infertility in months	63.59	44.53	59.24	35.18	0.180
Number of IUI before	1.03	1.27	1.09	1.38	0.602
Number of IVF before	1.32	1.23	1.35	1.23	0.784
Number of miscarriages	0.25	0.64	0.31	0.72	0.206

**Table 3:** Lifestyle and medical characteristics of investigated couples with infertility.

Parameters		Women		Men		Between-Group *p*
		Frequency	Percent	Frequency	Percent	
Employment position	unemployed	89	17.8	60	12.0	0.017
	outdoors	39	7.8	120	24.0	
	indoors	373	74.5	321	64.0	
Occupational hazards	no risk	465	92.8	453	90.4	0.172
	has risk	36	7.2	48	9.6	
Smoking tobacco	no	389	77.6	362	72.3	0.363
	yes	112	22.4	139	27.7	
Alcohol drinking	no	378	75.4	267	53.3	0.001
	sometimes	120	24.0	227	45.3	
	regularly	3	0.6	7	1.4	
Carbonated drinks	no	375	74.9	316	63.1	0.047
	yes	126	25.1	185	36.9	
Energy drinks	no	497	99.2	494	98.6	0.001
	yes	4	0.8	7	1.4	
Vitamins	no	360	71.9	416	83.0	0.001
	yes	141	28.1	85	17.0	
Physical activity	not regular	442	88.2	429	85.6	0.223
	regular	59	11.8	72	14.4	
Chronic illnesses	no	392	78.2	470	93.8	0.001
	yes	109	21.8	31	6.2	
Positive family history	no	262	52.3	298	59.5	0.022
	yes	239	47.7	203	40.5	

**Table 4:** Lifestyle and medical characteristics of examined patients according to ART outcome.

Parameters		No Pregnancy		Clinical Pregnancy		Between-Group *p*
		Frequency	Percent	Frequency	Percent	
Employment position	unemployed	115	14.7	34	15.3	0.878
	outdoors	124	15.9	35	15.8	
	indoors	541	69.4	153	68.9	
Occupational hazards	no risk	714	91.5	204	91.9	0.867
	has risk	66	8.5	18	8.1	
Smoking tobacco	no	587	75.3	164	73.9	0.651
	yes	193	24.7	58	26.2	
Alcohol drinking	no	515	66.0	130	58.6	0.066
	sometimes	259	33.2	88	39.6	
	regularly	6	0.8	4	1.8	
Carbonated drinks	no	542	69.5	149	67.1	0.501
	yes	238	30.5	73	32.9	
Energy drinks	no	772	99.0	219	98.6	0.681
	yes	8	1.0	3	1.4	
Vitamins	no	620	79.5	156	70.3	0.004
	yes	160	20.5	66	29.7	
Physical activity	not regular	685	87.8	186	83.8	0.116
	regular	95	12.2	36	16.2	
Chronic illnesses	no	665	85.3	197	88.7	0.187
	yes	115	14.7	25	11.3	
Positive family history	no	434	55.6	126	56.8	0.768
	yes	346	44.4	96	43.2	

**Table 5:** Lifestyle characteristics of couples with infertility and outcome of current ART cycle.

Parameters		Overall Sample		No Pregnancy		Clinical Pregnancy		Between-Group *p*
		Number	Percent	Number	Percent	Number	Percent	
Employment position	both unemployed	29	5.8	23	5.9	6	5.4	0.783
	one and one	212	42.3	163	41.8	49	44.1	
	both indoors	260	51.9	204	52.3	56	50.5	
Occupational hazards	both no risk	420	83.8	325	83.3	95	85.6	0.616
	one and one	78	15.6	64	16.4	14	12.6	
	both risk	3	0.6	1	0.3	2	1.8	
Smoking tobacco	both nonsmokers	302	60.3	236	60.5	66	59.5	0.714
	one and one	146	29.1	115	29.5	31	27.9	
	both smoke	53	10.6	39	10.0	14	12.6	
Alcohol	both non-drinkers	242	48.3	198	50.8	44	39.6	0.048
	one and one	161	32.1	119	30.5	42	37.8	
	both sometimes	98	19.6	73	18.7	25	22.5	
Carbonated drinks	both non-drinkers	266	53.1	210	53.8	56	50.5	0.532
	one and one	159	31.7	122	31.3	37	33.3	
	both drink	76	15.2	58	14.9	18	16.2	
Energy drinks	both non-drinkers	490	97.8	382	97.9	108	97.3	0.680
	one and one	11	2.2	8	2.1	3	2.7	
	both drink	0	0	0	0	0	0	
Vitamins	neither uses	334	66.7	271	69.5	63	56.8	0.011
	one and one	108	21.6	78	20.0	30	27.0	
	both use	59	11.8	41	10.5	18	16.2	
Physical activity	both not regular	387	77.2	308	79.0	79	71.2	0.094
	one and one	97	19.4	69	17.7	28	25.2	
	both regular	17	3.4	13	3.3	4	3.6	

**Table 6:** Correlations of lifestyle and occupational factors in the overall sample of women and men undergoing ART. First row per item—Spearman correlation coefficient; second row per item—*p* value; bold—significant.

Parameters	BMI	Work Position	Environs Hazards	Smoking	Alcohol	Sparkling Drinks	Energy Drinks	Vitamins	Physical Activity	Illnesses
Age	**0.090**	0.008	0.062	−0.019	−0.047	−0.061	−0.042	0.022	−0.022	−0.007
	**0.004**	0.798	0.051	0.548	0.141	0.053	0.185	0.494	0.490	0.829
BMI		**−0.064**	**0.082**	−0.006	**0.134**	**0.150**	0.039	**−0.144**	0.015	**0.119**
		**0.042**	**0.009**	0.843	**0.001**	**0.001**	0.220	**0.001**	0.626	**0.001**
Work position			0.023	**−0.089**	−0.004	−0.030	0.022	**0.126**	**0.068**	−0.024
			0.466	**0.005**	0.891	0.335	0.492	**0.001**	**0.032**	0.443
Environs hazards				0.016	**−0.083**	0.007	0.003	−0.034	−0.021	−0.008
				0.611	**0.008**	0.819	0.932	0.282	0.503	0.809
Smoking					0.054	**0.081**	−0.039	−0.048	**−0.067**	−0.021
					0.085	**0.010**	0.220	0.131	**0.033**	0.515
Alcohol						0.053	**0.063**	0.048	0.059	**−0.065**
						0.096	**0.047**	0.128	0.063	**0.040**
Sparkling drinks							**0.095**	−0.058	0.021	**−0.084**
							**0.003**	0.069	0.499	**0.008**
Energy drinks								−0.011	0.016	0.013
								0.727	0.614	0.686
Vitamins									**0.074**	**0.079**
									**0.019**	**0.013**
Physical activity										0.023
										0.467

**Table 7:** Regression model for clinical pregnancy in ART cycle for women and men separately assessed.

Models	Parameters	B Coefficient	Wald Coefficient	*p*	Odds Ratio (OR)	Confidence Interval OR Lower	Confidence Interval OR Upper
Women	Age	−0.075	8.941	0.003	0.928	0.884	0.975
	BMI	−0.022	0.353	0.552	1.022	0.951	1.099
	Illnesses	−0.093	0.114	0.735	0.911	0.532	1.561
	Employment	−0.050	0.115	0.735	0.952	0.715	1.268
	Occupation	−0.543	1.143	0.285	0.581	0.214	1.573
	Smoking	0.241	0.824	0.364	1.272	0.757	2.138
	Alcohol	0.030	0.054	0.817	1.030	0.801	1.325
	Carbon drink	0.198	0.606	0.436	1.219	0.740	2.009
	Energy drink	1.291	1.518	0.218	3.635	0.467	18.318
	Vitamins	0.882	12.910	0.001	2.416	1.493	3.908
	Activity	0.134	0.157	0.692	1.143	0.590	2.214
	Constant	0.679	0.286	0.593	1.971		
Men	Age	−0.047	5.270	0.022	0.954	0.916	0.993
	BMI	−0.020	0.404	0.525	0.981	0.923	1.042
	Illnesses	−1.467	3.920	0.048	0.231	0.054	0.985
	Employment	−0.046	0.083	0.774	0.955	0.698	1.307
	Occupation	0.487	1.820	0.177	1.627	0.802	3.301
	Smoking	−0.011	0.002	0.963	0.989	0.608	1.606
	Alcohol	0.143	1.620	0.203	1.154	0.926	1.439
	Carbon drink	−0.029	0.015	0.902	0.972	0.617	1.532
	Energy drink	−0.580	0.271	0.603	0.560	0.063	4.984
	Vitamins	0.179	0.384	0.536	1.196	0.679	2.104
	Activity	0.441	2.211	0.137	1.554	0.869	2.778
	Constant	0.990	0.633	0.426	2.692		

**Table 8:** Regression model for clinical pregnancy in ART cycle for couples.

Models	Parameters	B Coefficient	Wald Coefficient	*p*	Odds Ratio (OR)	Confidence Interval OR Lower	Confidence Interval OR Upper
Women and men	Gender	−0.091	0.213	0.644	0.913	0.620	1.344
	Age	−0.056	12.260	0.001	0.946	0.917	0.976
	BMI	−0.007	0.101	0.751	0.993	0.949	1.039
	Illnesses	−0.347	2.004	0.157	0.707	0.437	1.143
	Employment	−0.065	0.378	0.539	0.937	0.761	1.154
	Occupation	0.081	0.082	0.775	1.085	0.621	1.894
	Smoking	0.099	0.311	0.577	1.104	0.780	1.564
	Alcohol	0.109	1.721	0.190	1.115	0.948	1.312
	Carbon drink	0.059	0.122	0.727	1.061	0.761	1.479
	Energy drink	0.171	0.060	0.806	1.187	0.303	4.646
	Vitamins	0.547	9.237	0.002	1.729	1.215	2.460
	Activity	0.280	1.641	0.200	1.324	0.862	2.032
	Constant	1.025	0.948	0.330	2.786		
Behavior of couples	Employment	−0.142	0.594	0.441	0.868	0.604	1.245
	Occupation	0.040	0.020	0.889	1.041	0.597	1.815
	Smoking	0.077	0.226	0.635	1.080	0.787	1.481
	Alcohol	0.198	1.998	0.158	1.219	0.926	1.604
	Carbon drink	0.081	0.286	0.593	1.084	0.806	1.459
	Energy drink	0.162	0.054	0.817	1.175	0.300	4.607
	Vitamins	0.347	5.169	0.023	1.414	1.049	1.907
	Activity	0.225	1.235	0.266	1.253	0.842	1.864
	Constant	−1.536	21.293	0.001	0.215		

## Data Availability

The original contributions presented in this study are included in the article. Further inquiries can be directed to the corresponding author.

## Abbreviations

The following abbreviations are used in this manuscript: BMI—body mass index; ART—techniques of assisted reproduction; IUI—intrauterine insemination; IVF—in vitro fertilization; ICSI—intracytoplasmic sperm injection.
